# Prevalence and factors associated with burnout among frontline primary health care providers in low- and middle-income countries: A systematic review

**DOI:** 10.12688/gatesopenres.12779.3

**Published:** 2018-06-11

**Authors:** Sagar Dugani, Henrietta Afari, Lisa R. Hirschhorn, Hannah Ratcliffe, Jeremy Veillard, Gayle Martin, Gina Lagomarsino, Lopa Basu, Asaf Bitton

**Affiliations:** 1 Ariadne Labs, Brigham and Women’s Hospital and Harvard T.H. Chan School of Public Health, Boston, USA; 2 University of Toronto, Toronto, Canada; 3 Division of Hospital Internal Medicine, Mayo Clinic, Rochester, MN, USA; 4 Department of Medicine , Massachusetts General Hospital and Harvard Medical School, Boston, USA; 5 Feinberg School of Medicine, Northwestern University, Chicago, USA; 6 The World Bank Group, Washington, D.C., USA; 7 Institute of Health Policy, Management, and Evaluation, University of Toronto, Toronto, Canada; 8 The World Bank Group, Dar es Salaam, Tanzania; 9 Results for Development Institute, Washington, D.C., USA; 10 Armstrong Institute for Patient Safety & Quality, Johns Hopkins University, Baltimore, USA; 11 Division of General Medicine and Primary Care, Brigham and Women’s Hospital, Boston, USA; 12 Department of Health Care Policy, Harvard Medical School, Boston, USA

**Keywords:** primary health care; burnout;

## Abstract

**Background: **Primary health care (PHC) systems require motivated and well-trained frontline providers, but are increasingly challenged by the growing global shortage of health care workers. Burnout, defined as emotional exhaustion, depersonalization, and low personal achievement, negatively impacts motivation and may further decrease productivity of already limited workforces. The objective of this review was to analyze the prevalence of and factors associated with provider burnout in low and middle-income countries (LMICs).

**Methods: **We performed a systematic review of articles on outpatient provider burnout in LMICs published up to 2016 in three electronic databases (EMBASE, MEDLINE, and CAB). Articles were reviewed to identify prevalence of factors associated with provider burnout.

**Results: **A total of 6,182 articles were identified, with 20 meeting eligibility criteria. We found heterogeneity in definition and prevalence of burnout. Most studies assessed burnout using the Maslach Burnout Inventory. All three dimensions of burnout were seen across multiple cadres (physicians, nurses, community health workers, midwives, and pharmacists). Frontline nurses in South Africa had the highest prevalence of high emotional exhaustion and depersonalization, while PHC providers in Lebanon had the highest reported prevalence of low personal achievement. Higher provider burnout (for example, among nurses, pharmacists, and rural health workers) was associated with high job stress, high time pressure and workload, and lack of organizational support.

**Conclusions: **Our comprehensive review of published literature showed that provider burnout is prevalent across various health care providers in LMICs. Further studies are required to better measure the causes and consequences of burnout and guide the development of effective interventions to reduce or prevent burnout.

## Introduction

Primary health care (PHC) includes provision of services for the prevention, treatment, management, rehabilitation, and palliation of disease, and is integral to achieving global health security, universal health coverage and the Sustainable Development Goals ^1– 7^. A robust PHC system requires an adequate number of trained and motivated health care providers ^6, 8, 9^. Alarmingly, the World Health Organization (WHO) has estimated that the global shortage of providers will increase by 80% to 12.9 million over the next 20 years and has called for the development of an expanded, high-quality workforce ^10^. Given the projected shortages, there is great interest in strategies to retain existing providers and improve provider efficiency and productivity. Further, a positive work environment will reduce workforce turnover, and improve quality of life and care. The reasons for lower efficiency and productivity are unclear, and may be linked to extrinsic motivational factors including financial and non-financial, organizational, and environmental incentives ^11^ or to incompletely described intrinsic motivational factors such as achievement, recognition, responsibility, and growth ^12^, which may be negatively impacted by provider burnout.

Burnout, as described by Freudenberg ^13^ and expanded by Maslach, is comprised of three dimensions: emotional exhaustion (‘emotionally overextended and exhausted by […] work’), depersonalization (‘unfeeling and impersonal response towards recipients of one’s care or service’), and low personal achievement (‘feelings of competence and successful achievement […] with people’), and results in negative work experiences ^14– 16^. There are a number of surveys used to assess burnout ^17, 18^; however, the Maslach Burnout Inventory (MBI) has emerged as perhaps the most widely used survey to assess burnout across a wide variety of work and cultural settings ^19^. Studies using the MBI in the United States, Canada, and mostly high-income countries in Europe have found that up to half of outpatient providers report high levels of emotional exhaustion, depersonalization, and a sense of low personal achievement ^20– 22^). These findings are supported by a systematic review which documented high levels of burnout in both outpatient and inpatient providers in high-income countries ^23^. In these studies, high burnout was associated with feeling undervalued and unsupported, having too much paperwork, and the existence of long waits for specialists and tests, among other factors ^20– 22^.

Identifying and characterizing burnout is important as it can have a negative impact on providers and patient care. Studies from predominantly high-income countries have shown that provider burnout is associated with adverse events including medical errors, unexplained work absenteeism, reduction in quality of care ^24, 25^, higher number of negative rapport-building statements (physician or patient offers a statement ‘characterized as criticism or disagreement’) ^26^, job dissatisfaction ^27^, and poor patient satisfaction ^28, 29^. A large study of 11,530 health professionals in Spain and Latin America showed that higher emotional exhaustion was associated with higher absenteeism, intention to exit the profession, and low quality of personal and family life ^30^.

Despite the growing recognition of the need to retain trained providers and improve the quality of care they provide, there is no comprehensive analysis of the burden of provider burnout in low and middle-income countries (LMICs). In addition to the paucity of such syntheses, current data are cross-sectional without an evaluation of potential change over time and most studies have not characterized institutional (e.g., institutional management, quality, or supervision), individual, socioeconomic, or geopolitical factors that could potentially contribute to provider burnout. To address this gap, we conducted a systematic review to describe the prevalence of and factors associated with outpatient provider burnout in LMICs. These findings may help managers and policymakers develop and implement effective interventions to prevent burnout and improve work productivity, efficiency, quality and retention.

## Methods

### Data sources and search strategy

We performed a systematic literature search to identify articles on burnout among outpatient health care providers in LMICs. The review protocol was not registered on an online portal. We focused on outpatient care settings as this is where the majority of primary health care services are provided. Our initial search was based on articles published in EMBASE (from 1947), MEDLINE (from 1966), and Commonwealth Agricultural Bureau (CAB) Abstracts (from 1973) up to December 1, 2014. We developed a broad search strategy for each key term: ‘burnout’, ‘healthcare providers’, and ‘LMICs’, through a combination of text words, words in the abstract or title, and Medical Subject Headings (MeSH). For burnout, we included “motivation” and “achievement”. For healthcare providers, we included “physician”, “nurse”, and “community health worker”; and for LMICs, we included “developing countries”, “resource constrained”, and “resource poor”. We used the World Bank system to classify countries as low or middle-income based on gross national income per capita ^31^. The search terms were combined using ‘AND’ to identify articles that included all three concepts, as outlined in the S1 Supplementary Material. The search was updated using the same methodology to include articles from December 1, 2014 through January 23, 2016.

### Study selection

The titles and abstracts were reviewed independently by two authors. Research articles written in English were included if the study was based in an LMIC and explicitly investigated *burnout* and not solely work-related depression, anxiety, or stress, in outpatient healthcare workers. Articles were excluded if they were conference abstracts, case reports, case series, simulations, review articles, editorials, commentaries, perspectives, personal narratives, or qualitative studies; if the full-length article was not available; if the study had fewer than 50 subjects; or if the study focused on trainees (for example, students, residents, or fellows), inpatient providers, or on veterinary care providers. Discrepancies between the authors in abstracting data were resolved by discussion or through consultation with other authors. The detailed selection strategy is outlined in Supplementary File 3.

### Data extraction and analysis

We collected information on the study location and design, participant demographics, cadre, and duration in practice. For burnout, we collected information on the type of burnout inventory used, and estimates of overall burnout and its subcomponents (depersonalization, emotional exhaustion, and level of personal achievement).

## Results

### Study characteristics

Our initial search (on December 1, 2014) generated 5,412 articles (2,046 from EMBASE, 847 from CAB Abstracts, and 2,519 from MEDLINE), of which 735 were duplicates. Using eligibility criteria described above, 11 articles were included in final data extraction and analysis ( Supplementary File 3). We updated the search on January 23, 2016; we identified 770 articles (total of 6,182 articles when combined with search on December 1, 2014) and identified 9 additional articles that met our eligibility criteria ( S2 Supplementary Material). The 20 studies included in the final analysis spanned all global regions, and focused on various providers including physicians, pharmacists, nurses, community health workers, and midwives. Only two studies were based in low income countries while the rest were based in middle-income countries.

Across the reported studies, the mean age of healthcare providers ranged from 26.4 years to 47.4 years ( Table 1). Studies included a range of provider types including HIV service providers (3 studies), PHC and general practitioners (five studies) and community-based workers (six studies). Cadres included physicians, nurses and midwives, dentists, pharmacists, community health workers and health volunteers. The range of education varied based on cadre, with lower rates among community health workers and volunteers compared to providers with a formal degree. For example, among AIDS volunteers in South Africa, 93.7% had completed secondary or high school education while only 2.4% had ‘higher education’ ^32^, whereas among HIV caregivers in Brazil, 52.9% of volunteers had a university level education ^33^.

**Table 1.** Characteristics of outpatient healthcare providers. All studies used the Maslach Burnout Inventory, except as follows: Kruse ^50^ (single question validated against a full occupational burnout scale); Akintola ^32^ (modified MBI score); Jocic ^51^ (Self-assessment test with 15 questions assessed on a Freudenberg scale); Muliira ^48^ (Professional Quality of Life Scale); Pandey ^49^ (Copenhagen Burnout Inventory).

Author, year	Country (World Bank Region)	Type of Healthcare Provider	Sample Size	Sex, % participants	Age, years ^a^	Position/Type of work, % participants	Number of years in present position or occupational tenure, % participants ^b^
Benevides- Pereira, 2007	Brazil (Latin America and the Caribbean)	HIV Healthcare Providers	87	Male, 18.4% Female, 79.3% Not Reported, 2.3%	36.4 ± 9.5	Voluntary, 63.2% Not voluntary, 36.8%	>5, 73.5% ˃5, 25.3% Not Reported, 1.2%
da Silva, 2008	Brazil (Latin America and the Caribbean)	Community- based health agents	141	Male, 7.8% Female, 92.2%	38.9 ± 11.4	Not Reported	≤3.5, 51.1% >3.5, 48.9%
Engelbrecht, 2008	South Africa (Sub-Saharan Africa)	Nurses	543	Not Reported	Not Reported	Not Reported	Not Reported
Kruse, 2009	Zambia (Sub-Saharan Africa)	HIV Healthcare Providers	483	Female, 86.6% Not Reported, 13.4%	Median,37 (31–45)	Physicians, 1.5% Clinical officers, 10.8% Nurses, 50.5% Midwifes, 27.9% Pharmacy technicians, 4.1% Others, 5.2%	Median (IQR) ^d^ 10 (4–17)
Putnik, 2011	Serbia (Europe and Central Asia)	Primary Healthcare Physicians	373	Male, 16.0% Female, 84.0%	Male, 47.4 ±10.2 Female, 47.4 ± 8.5	Not Reported	Mean Male, 19.8% Female, 19.6%
Ge, 2011	China (East Asia and Pacific)	Community Health Workers	1694	*City of* *Shenyang ^c^: City* *of Benxi ^c^* Male, 22.2% Male, 15.8% Female, 77.8% Female, 84.2%	Median ≥ 40	*City of* *Shenyang City of* *Benxi* Physicians, 56.6% Physicians, 40.4% Nurses, 35.4% Nurses, 46.6% Others, 7.9% Others, 13.0%	Not Reported
Malakouti, 2011	Iran (Middle East and North Africa)	Rural Health Workers	227	Male, 29.9% Female, 70.1%	35.1 ± 7.2	Not Reported	Mean ± SD 12.0 ± 7.6
Calgan, 2011	Turkey (Europe and Central Asia)	Community Pharmacists	251	Male, 41.4% Female, 58.6%	42.1 ± 11.2	Not Reported	<10, 43.4% 10–19, 25.7% 20–29, 21.2% ≥ 30, 9.6%
Alameddine, 2012	Lebanon (Middle East and North Africa)	Primary Healthcare Providers	755	Male, 49.6% Female, 50.3% Not Reported, 0.1%	Median, 36–45	Generalists (including dentists), 23% Medical Specialists, 21.7% Nurses, 32.7% Allied health professionals, 15.1% Other health professionals, 7.4%	≤5, 61.8% 6–10, 19.2% <10, 16.1% Not Reported, 2.9%
Akintola, 2013	South Africa (Sub-Saharan Africa)	AIDS Volunteer Caregivers	126	Male, 100%	35.0 ± 7.1	Care of HIV/AIDS patients, 36.8% Care of orphans, 14.4% Care of both groups, 48.8%	Mean ± SD 6.8 ± 2.1
Jocic, 2014	Serbia (Europe and Central Asia)	Community Pharmacists	647	Male, 24.9% Female, 75.1%	Median, 41–50	Not Reported	≤5, 16.7% 6–10, 27.0% >10, 56.3%
Karakose, 2014	Turkey (Europe and Central Asia)	General practitioners	71	Male, 87.3% Female, 12.7%	<30 years, 29.6% 31–45 years, 54.9% ≥46 years, 15.5%	Not Reported	Not Reported
Ding, 2014	China (East Asia and Pacific)	Community Health Center providers	1243	Not reported	Not reported	Not reported	≤10, 28.7% 11–20, 31.4% 21–30, 26.4% >30, 13.5%
Cagan, 2015	Turkey (Europe and Central Asia)	Primary Healthcare Providers	418	Male, 33.3% Female, 66.7%	36.6 ± 6.3	Physicians, 44.4% Nurses, 25.4% Midwives, 30.1%	Not Reported
Cao, 2015	China (East Asia and Pacific)	Community Health Nurses	485	Female, 100%	26.4 ± 3.8	Staff nurses, 94.2% Head nurses, 5.8%	≤5, 57.9% 6–10, 29.1% >10, 13.0%
Silva, 2015	Brazil (Latin America and the Caribbean)	Primary Healthcare Providers	194	Male, 16.5% Female, 83.5%	44.9 ± 10.5	Physicians, 27.8% Nurses, 37.1% Dentists, 20.1% Social assistants, 14.9%	Not Reported
Muliira, 2015	Uganda (Sub-Saharan Africa)	Midwives	224	Male, 20.5% Female, 79.5%	34 ± 6.3	Antenatal clinic, 43.3% Delivery ward or labour room, 33.0% Postnatal ward, 23.7% Health Center Level II 49.1% Health Center Level III 33.5% Health Center Level IV 17.4%	3 ± 1.3
Hu, 2015	China (East Asia and Pacific)	Nurses	420	Female, 100%	≤30 years, 48.3% 31–40 years, 34.5% ≥41 years, 17.2%	Nurse, 41.4% Senior Nurse, 24.8% Chief Nurse or higher, 33.8%	≤3 years, 29.3% 4–10 years, 22.4% ≥11 years, 48.3%
Pandey, 2015	India (South Asia)	Accredited Social Health Activists	177	Female, 100%	31.9 ± 6.7	Accredited Social Health Activists, 100%	Not reported
Cao, 2016	China (East Asia and Pacific)	Community Health Nurses	456	Male, 4.7% Female, 95.4%	34.1 ± 7.1	Community health nurses, 100%	1–5, 5.5% 6–10, 24.6% 11–15, 38.3% 16–20, 26.8% >20, 4.8%

Key:

^a^Age (in years) is reported as mean ± standard deviation, except where noted

^b^Number of years in service (in %) except where noted

^c^Shenyang, Benxi are two cities in Liaoning Province in northeast China. Shenyang has 7.2 million inhabitants, and Benxi has 3.1 million inhabitants

^d^IQR : interquartile range

### Maslach Burnout Inventory (MBI) to measure provider burnout

The MBI is the most widely used inventory to assess burnout, and consists of 22 questions across three dimensions: emotional exhaustion (nine questions), depersonalization (five questions), and personal achievement (eight questions). Each question is scored on a scale from 0 (never) to 6 (everyday). The points from each dimension are added to provide a total score for that dimension. The score for each dimension can be categorized as low, moderate, or high: emotional exhaustion (low ≤ 13; moderate 14 to 26; high ≥ 27); depersonalization (low ≤ 5; moderate 6 to 9; high ≥ 10); and personal achievement (high ≤ 33; moderate 34 to 39; low ≥ 40) ^22^. Higher scores on emotional exhaustion and depersonalization, and a lower score on personal achievement, are associated with higher provider burnout. The development, reliability, and validity of the MBI have been previously described ^15^. Of the 20 studies, 15 used the MBI ^33– 47^, one ^32^ used a modified version of MBI, and four ^48– 51^ used other assessment tools ( Table 2).

**Table 2.** Prevalence of burnout among outpatient healthcare providers.

Author, year	Emotional Exhaustion (%) ^a^ (Mean Score ± SD)	Depersonalization (%) ^a^ (Mean Score ± SD)	Personal Achievement (%) ^a^ (Mean Score ± SD)	Other Results
Benevides-Pereira, 2007	Low, 40.2% Moderate, 33.3% High, 26.4% (19.1 ± 10.3)	Low, 56.3% Moderate, 26.4% High, 17.2% (4.2 ± 5.4)	Low, 40.2% Moderate, 40.2% High, 19.5% (39.6 ± 7.2)	-
da Silva, 2008	Moderate or High, 70.9%	Moderate or High, 34.0%	Moderate or High, 47.5%	Report of aspects related to burnout, 84.4% Burnout by MBI criteria, 24.1%
Engelbrecht, 2008	Low, 0.2% Moderate, 30.9% High, 68.7% (31.3 ± 9.3)	Low, 1.8% Moderate, 12.9% High, 85.1% (17.8 ± 5.0)	Low, 0.7% Moderate, 91.0% High, 8.3% (20.3 ± 6.8)	-
Kruse, 2009	Not Reported	Not Reported	Not Reported	No Burnout, 6.9% Stress without Burnout, 42.0% Occasional Burnout, 23.3% Burnout not improving, 4.3% Severe Burnout, 23.5%
Putnik, 2011	Male Low: 22.4% Moderate: 36.2% High: 41.4% (2.3 ± 1.3) Female Low: 17.0% Moderate: 33.7% High: 49.4% (2.5 ± 1.3)	Male Low: 48.3% Moderate: 46.6% High: 5.2% (0.7 ± 0.7) Female Low: 55.4% Moderate: 30.1% High: 14.4% (0.8 ± 0.9)	Male Low: 10.3% Moderate: 15.5% High: 74.1% (5.1 ± 1.1) Female Low: 4.2% Moderate: 17.3% High: 78.5% (5.1 ± 5.2)	-
Ge, 2011	*City of Shenyang* (7.2 ± 5.5) *City of Benxi* (6.9 ± 5.8)	*City of Shenyang* (3.6 ± 4.3) *City of Benxi* (3.3 ± 4.4)	*City of Shenyang* (24.4 ± 10.8) *City of Benxi* (25.4 ± 10.5)	-
Malakouti, 2011	Low, 72.6% Moderate, 15.1% High, 12.3% (14.5 ± 9.9)	Low, 86.7% Moderate, 8.0% High, 5.3% (2.2 ± 3.4)	Low, 43.7% Moderate, 19.0% High, 37.4% (33.8 ± 10.4)	-
Calgan ^b^, 2011	Moderate, 27.1% ^c^ High, 1.2% (16.8 ± 6.3)	Moderate, 13.9% ^c^ High, 0.8% (Mean 4.0, Range 0–14)	Moderate, 24.7% ^c^ High, 71.3% (Mean 22.0, Range 9–32)	-
Alameddine, 2012	Low, 59.1% Moderate, 17.7% High, 23.2%	Low, 70.7% Moderate, 15.5% High, 13.8%	Low, 64.9% Moderate, 16.4% High, 8.7%	-
Akintola, 2013	Not Reported	High, 50% (8.5 ± 1.6)	High, 60% (8.9 ± 1.2)	-
Jocic, 2014	Not Reported	Not Reported	Not Reported	No Burnout, 37.1% Risk for Burnout, 9.0% Before Burnout, 9.6% Burnout, 29.7% Combustion, 14.7%
Karakose, 2014	Male (mean 2.8 ± 1.2) Female (mean 3.4 ± 1.1)	Male (mean 2.5 ± 1.0) Female (mean 2.5 ± 1.1)	Male (mean 4.0 ± 1.0) Female (mean 3.9 ± 0.8)	-
Ding, 2014	Mean (10.1 ± 6.5)	Mean (5.7 ± 5.2)	Mean (24.1 ± 9.3)	-
Cagan, 2015	Male (median 14.0) Female (median 24.0)	Male (median 4.0) Female (median 3.0)	Male (median 15.0) Female (median 23.0)	-
Cao, 2015	Mean (27.0 ± 10.6)	Mean (8.4 ± 7.0)	Mean (25.7 ± 9.3)	-
Silva, 2015	Low, 36% Average, 21% High, 43%	Low 51% Average, 33% High, 17%	Low, 32% Average, 43% High, 25%	Burnout risk: High 27.8% Medium 26.3% Low 45.9%
Muliira, 2015	Not reported	Not reported	Not reported	Low level of burnout, 0% (male), 1.8% (female) Average level of burnout 17.0% (male), 71.0% (female) High level of burnout 2.2% (male), 8.0% (female)
Hu, 2015	≤30 years, mean (12.1 ± 5.3) 31–40 years, mean (14.2 ± 5.6) ≥41 years, mean (13.6 ± 6.3)	≤30 years, mean (16.1 ± 7.3) 31–40 years, mean (15.3 ± 6.7) ≥41 years, mean (14.6 ± 5.7)	≤30 years, mean (22.1 ± 7.5) 31–40 years, mean (20.9 ± 7.2) ≥41 years, mean (20.1 ± 6.5)	-
Pandey, 2015	Not reported	Not reported	Not reported	Mean burnout (4.0 ± 1.4)
Cao, 2016	26.5 ± 10.5	8.6 ± 6.5	24.7 ± 9.4	-

Key:

^a^Prevalence reported as percentage of participants with scores. Higher Emotional Exhaustion and Depersonalization, and lower Personal Accomplishment, are associated with higher burnout Low refers to low score, Moderate refers to moderate score, and High refers to high score

^b^values are relative to Hungarian national norms ^41^

*Emotional Exhaustion* was evaluated in 15 studies, and the average score ranged from 2.3 ^38^ to 31.3 ^37^. The lowest prevalence of moderate or high emotional exhaustion (score ≥14) was seen among rural health workers in Iran (27.4%) ^40^ and the highest was among nurses in South Africa (99.6%) ^37^. Eight studies reported the proportion of people with different levels of emotional exhaustion; of these, six studies showed moderate to high levels of emotional exhaustion were reported in more than one-third of healthcare providers studied.

*Depersonalization* was reported in 16 studies. Similar to emotional exhaustion, a high level was reported, with the average score ranging from 0.7 ^38^ to 17.8 ^37^. The lowest prevalence of ‘moderate or high’ depersonalization (score ≥6) was seen among rural health workers in Iran (13.3%) ^40^ and the highest among nurses in South Africa (98.0%) ^37^. Nine studies reported the proportion of people with different levels of depersonalization; of these, six studies showed moderate to high levels of depersonalization in more than one-third of healthcare providers.

*Personal Achievement* was reported in 16 studies, and the average score ranged from 3.9 ^43^ to 39.6 ^33^. The lowest prevalence of ‘moderate or high’ personal achievement (score ≤29) was seen among primary health care providers (25.1%) in Lebanon ^42^, whereas the highest (99.3%) was seen among nurses in South Africa ^37^.

### Non-MBI measures of provider burnout

Four studies used non-MBI tools to measure burnout among providers. A study based in Serbia used a self-assessment test (15 questions assessed on a Freudenberg scale) and reported that 44.4% of community pharmacists had high levels of burnout ^51^. A study based in Zambia used a single question to assess burnout and reported that 27.8% of HIV healthcare providers had burnout that was severe or not improving with time ^50^. In Uganda, Muliira and colleagues ^48^ used the Professional Quality of Life Scale, which classifies provider burnout levels into three categories: high, average, and low, and reported that 89.3% and 10.1% of female midwives had average and high levels of burnout, respectively, while 82.6% and 10.8% of male midwives had average and high levels of burnout, respectively. Pandey and colleagues used a modified Copenhagen Burnout Inventory (scale 1–7, with higher scores reflecting higher burnout) ^17^ and showed that accredited social health activists (ASHA) in India had a mean burnout score of 4.0 ± 1.4 ^49^. Further, one study in South Africa used a modified version of MBI, in which the emotional exhaustion domain was excluded. Using this modified version, Akintola and colleagues reported a high level of depersonalization and personal achievement among AIDS care volunteers ^32^.

### Variables associated with provider burnout

Seven studies investigated variables associated with overall burnout ( Table 3). Among HIV healthcare providers in Zambia, Kruse and colleagues ^50^ observed that the 36–45 year age group had a higher relative risk (1.5 [1.1–1.9], at 95% confidence interval) of burnout compared with those 45 years or older. In Serbia, Jocic and colleagues ^51^ found that burnout was more common among older community pharmacists (51–60 years) compared with their younger colleagues. In addition, Kruse and colleagues ^50^ showed that females (relative risk 2.0 [1.1–2.7]), providers who worked other jobs (relative risk 1.4 [1.1–1.6]) and providers who knew a co-worker who had quit work (relative risk 1.6 [1.2–2.0]) reported higher levels of burnout. Among rural health workers in Iran, provider burnout was associated with longer work experience, high job stress (70.1% in those with burnout versus 37.7% in those without burnout; p=0.001), and having a higher General Health Questionnaire score, a measure of higher psychological distress ^40^.

**Table 3.** Variables associated with provider burnout among studies using the Maslach Burnout Inventory and reporting these variables (15/20).

Author, year	Overall Burnout	Emotional Exhaustion	Depersonalization	Personal Achievement
Benevides- Pereira, 2007	Not Reported		Positive association: male sex	Positive association: younger age
da Silva, 2008		No significant associations identified	Positive association: being black; those absent from work once in the 30 days prior to the interview Inverse association: female sex; age 41 years or higher; monthly family income between 4 and 5, and above 7 minimum salaries; working where 20% or more users are of private medical care systems	Positive association: age =41 years
Engelbrecht, 2008		Positive association: availability of resources; time pressure of workload; conflict and social relations	Positive association: availability of resources; time pressure of workload	
Kruse, 2009	Positive association: female sex; age (36 to 45 years); working other jobs; knowing a co-worker who left	Not reported		
Putnik, 2011	None reported			
Ge, 2011		*Inverse association:* *intrinsic and extrinsic job* *satisfaction*	Positive association: intrinsic job satisfaction	
Malakouti, 2011 *	Positive association: longer work experience; higher GHQ scores; higher job stress	Not Reported		
Calganb, 2011		Positive association: lower age; lower work contentment; lower satisfaction with customers; excessive workload; excessive time pressure; higher frequency of work stress; fewer years in practice	Positive association: lower age; being unmarried; lower satisfaction with customers; excessive time pressure; higher frequency of work stress; fewer years in practice	Positive association: lower age; higher work contentment; higher satisfaction with customers; lower time pressure; lower frequency of work stress; more years in practice
Alameddine, 2012		Positive association: likelihood to quit job	Positive association: likelihood to quit job	*Inverse association:* *likelihood to quit job*
Akintola, 2013		Not Reported	Positive association: Type of volunteer and lack of support	Positive association: total stress; lack of support; overwhelming nature of the disease; difficulty dealing with distress and dying
Jocic, 2014	Positive association: higher age	Not Reported		
Karakose, 2014		*Inverse association: intrinsic* *job satisfaction.* No association with extrinsic job satisfaction, and general job satisfaction	No association with intrinsic job satisfaction, extrinsic job satisfaction, or general job satisfaction	Positive association: intrinsic job satisfaction, extrinsic job satisfaction, and general job satisfaction
Ding, 2014		Positive association: effort-reward ratio, over commitment, and anxiety symptoms *Inverse association: length* *of employment*	Positive association: effort-reward ratio, over commitment, and anxiety symptoms *Inverse association: length* *of employment*	Positive association: length of employment, and over commitment Inverse association: effort- reward ratio, and anxiety symptoms
Cagan, 2015	No relationship with gender, marital status, or profession. Personal accomplishment positively associated with working in districts. Emotional exhaustion positively associated with low perceived economic status and not personally choosing working department. Emotional exhaustion and depersonalization negatively associated with job happiness.			
Cao, 2015		*Inverse association: general self-concept, leadership, communication, knowledge,* *staff relationship, caring, affective commitment, normative commitment, continuance* *commitment*		
Silva, 2015	Positive association with risk of burnout ^$^: age >30 years, work week >40 hours, professional dissatisfaction, desire to abandon the profession, feeling of discomfort, reporting that work was not a source of realization, mental disorder diagnosed by a psychiatrist, emotional tension, and limited/average future expectations			
Muliira, 2015	Positive association: associate degree (compared to Bachelor’s or Masters’ degree), being married, and involvement in non- midwifery health care activities at work			
Hu, 2015	Positive association: constant term, unmarried status, junior college-level education, difficulties between doctor and nurse, difficulties between nurse and patient, and difficulties between nurse and nurse Inverse association: job satisfaction	Positive association: age >30 years, non-single marital status, associate/ bachelor degree/higher, being senior nurse/ charge nurse/higher, employment status (formal establishment), >3 years employment, job dissatisfaction, unfair/ inappropriate content of continuing education opportunities, difficulty with interpersonal relationships, income =1000 RMB	Positive association: job dissatisfaction, unfair/ inappropriate content of continuing education opportunities, difficulty with interpersonal relationships,	Positive association: single marital status, job dissatisfaction, unfair/ inappropriate content of continuing education opportunities, difficulty with interpersonal relationships
Pandey, 2015	Positive association with “deep emotional labor”, or altering felt emotions to match expections *Inverse association:* *job satisfaction and* *“surface emotional* *labor”, or altering* *expressed (but not* *felt) emotions to* *match expectations*			
Cao, 2016		*Inverse association: perceived organization support, general self-concept, leadership,* *communication, knowledge, staff relationship, and caring*		

Higher Emotional Exhaustion and Depersonalization, and lower Personal Accomplishment, are associated with higher burnout Key:

^*^GHQ: General Health Questionnaire; higher scores indicate higher psychological distress; Job stress based on Steinmentz test ^40^

^$^High risk of burnout: (high emotional exhaustion + high depersonalization + high professional realization) OR (high emotional exhaustion + low depersonalization + low professional realization) OR (low emotional exhaustion + high depersonalization + low professional realization); moderate risk of burnout: high emotional exhaustion OR high depersonalization OR low professional realization; low risk of burnout: (low emotional exhaustion + low depersonalization + high professional realization) RMB: Renminbi or Yuan (currency of China) Emotional labor: “the process of regulating both feelings and expressions for the organizational goals”. Surface-level emotional labor is showing fake emotions and deep-level emotional labor is done when providers “alter their felt emotions genuinely to match the ones desired by the organization.

Twelve studies in our analysis reported on factors associated with specific dimensions of burnout. Higher rates of e *motional exhaustion* were associated with higher time pressure of workload and excessive workload ^37, 41^. In Turkey, among pharmacists who reported having “excessive” time pressure, the mean emotional exhaustion score was higher (19.2 ± 5.9) compared to those with low time pressure (10.7 ± 5.6, p<0.001) ^41^. Higher emotional exhaustion scores were also seen in pharmacists who had less work experience (10 years or less [17.6 ± 5.7]) compared to those who had worked longer in the field (30 years or more [13.8 ± 6.9]; p=0.007) ^41^. In community health workers in China, emotional exhaustion was associated with lower intrinsic and extrinsic job satisfaction ( Table 3) ^39^. Intrinsic satisfaction evaluates job-related tasks (e.g. professional development opportunities) while extrinsic satisfaction evaluates aspects external to the job (e.g. wages, benefits and bonuses) ^39^. In general, inverse associations were seen with emotional exhaustion and perceived organizational support, leadership, and staff relationships.

Variables associated with *depersonalization* were evaluated in 12 studies. As seen with emotional exhaustion, higher levels of depersonalization were associated with excessive time pressure and lack of support ^32, 37, 41^. Amongst pharmacists in Turkey with excessive time pressure, the median depersonalization score was higher compared to those with low time pressure (4 versus 1, p=0.004) ^41^. Similar findings were seen among nurses providing HIV care in South Africa ^37^. Among AIDS care volunteers in South Africa, higher depersonalization was seen among those who perceived a ‘lack of support’ (p=0.025) ^32^. In other studies, higher depersonalization was seen among men compared with women and was associated with higher rates of recent absenteeism (odds ratio 3.0 [1.2–7.8], p=0.02) ^33, 36^. Lower rates of depersonalization were associated with overall higher intrinsic and extrinsic job satisfaction among community health workers in China ^39^.

Consistent with trends observed for emotional exhaustion and depersonalization, higher personal achievement was associated with lower time pressure, lower stress, and higher availability of resources and intrinsic job satisfaction ^32, 37, 39, 41^. For example, among nurses in South Africa, higher personal achievement was significantly associated with lower time pressure ^37^, and among AIDS care volunteers in South Africa, lower personal achievement was associated with higher rating of ‘lack of support’ (p=0.03), ‘professional uncertainty’ (p=0.008), and overwhelming nature of their patients’ disease (p=0.04). One study showed that higher emotional exhaustion was associated with increased likelihood of quitting the job (odds ratio 3.46 [2.00–5.99], p<0.001) while higher personal achievement lowered that risk (odds ratio 3.05 [1.67–5.56], p<0.001) ^42^.

## Discussion

In this systematic literature review, we observed that burnout is prevalent across a range of frontline PHC service delivery providers including physicians, nurses, pharmacists, and community health workers in various outpatient health care settings including HIV care clinics in a number of LMICs. To our knowledge, this is the first systematic review to describe provider burnout in LMICs, and provides insight into factors that could influence worker productivity, efficiency, quality, and retention through their influence on burnout. The level of burnout across each MBI dimension is comparable to rates observed among outpatient general internists in the US, family doctors in Canada, and family doctors across 12 countries in Europe ^20– 22^. These studies, which include several high-income countries, found rates of high emotional exhaustion (range 43.0% to 48.1%), high depersonalization (32.7% to 46.3%), and low personal achievement (20.3% to 47.9%).

We were able to identify several consistent factors associated with different dimensions of burnout. Possibly modifiable factors included levels of organizational support, time pressure and workload, as well as the availability of accessible opportunities for professional growth, though the association of these factors with burnout is likely to depend on the cadre in question, their training level, degree of autonomy, and relationship with communities and patients. The roles and responsibilities of different cadres of health workers depends on the study, and therefore should be interpreted carefully. These results are generally supported by the other studies that showed positive association with longer work hours and inverse association with job satisfaction ^34, 35, 48, 49^. Absence of supportive supervision (managers helping health workers to do their job better) appears to also be related to the presence of burnout. Supportive supervision can provide health care workers with opportunities for new skills development as well as improving effectiveness and efficiency of their care delivery. While higher disease burden of patients is less amenable to simple solutions, service delivery changes such as multidisciplinary teams may provide approaches to reducing provider burnout. These reported findings were generally similar to findings seen in high-income countries ^20, 22^.

Additionally, there were also factors which were not modifiable through workplace interventions including age, gender, and level of education, which is also similar to results seen in high-income countries ^20, 22^. Further research is required to understand why burnout is higher among these groups in order to ensure that effective support and coping strategies are provided for health care workers.

Improving PHC will be critical for achieving universal health coverage and the Sustainable Development Goals by 2030. In LMICs, this will require an available, accessible, and acceptable workforce that can deliver efficient, high-quality patient care ^52^. While increasing the number of providers in some regions is clearly necessary, health systems will also have to focus on ways to retain existing staff by reducing burnout, providing a supportive environment, creating opportunities for personal achievement and growth, reducing stress and maintaining motivation. Interventions focused on improving interpersonal relationships, supportive work environments, supportive supervision including mentorship, coaching incentives and training on self-awareness and mindfulness, may help to reduce burnout, however evidence from LMICs is often lacking and the generalizability of many of these interventions done in high income settings is not certain ^53– 59^. For example, a pre-post study of 84 mental health professionals in the United States found that a one-day retreat and training focused on increasing knowledge of and strategies to prevent burnout was associated at six weeks with significant decreases in emotional exhaustion and depersonalization ^55^. While larger studies on effectiveness of interventions are generally lacking, a few ongoing studies in high-income countries on interventions to reduce burnout and improve patient outcomes may shed light on promising approaches, although these are generally limited to specific cadres or settings ^55, 60^. Further, provider depression may be a cause or consequence of provider burnout, and studies will be required to explore if they are separate or linked constructs.

Our paper has a number of important limitations. We included articles from three widely used electronic databases, and different cadres of health care providers across LMICs from many regions. However, we did not include articles that were not in English or articles that were published outside of the three search engines, including in non-peer reviewed literature. Further, we did not evaluate the quality of the studies. Only 15 of the 20 included studies used the MBI, and among them, differences in study population and design precluded analyses across different cadres of health providers.

We included a wide variety of primary care providers ranging in training from physicians to community health workers and volunteer caregivers. Because of high prevalence of HIV in some of the countries, we included providers of HIV services as they are a significant source of critical first contact care for people living with HIV/AIDS. The comparability of findings across these widely different health workers who operate within the primary health care sector in LMICs may not be complete. Finally, the quality of evidence available in the included studies was often low. Many studies relied on non-validated measures or used thresholds for defining burnout which were established for high-income countries but applied to LMIC. More work is needed to ensure high-quality measurement and the use of contextually appropriate diagnosis criteria. Additionally, further studies should ensure higher response rates among responders, describe the characteristics of non-responders, and clarify the level of anonymity responders have while participating in studies. Further, as many studies are cross-sectional, it will be challenging to generalize these findings to different contexts and situations. However, despite these limitations, we were able to observe consistent trends in burnout across these different health providers and in different countries.

Our understanding of factors related to high rates of burnout and low provider motivation in LMICs is still in its infancy. This review is based on 20 cross-sectional studies of diverse health providers in different countries. To better describe burnout and reduce its impact on provider retention and quality, further research should focus on more comprehensive investigation of the i) burden of provider burnout from diverse health care providers at different levels in the health care system, ii) demographic, socioeconomic, institutional, and geopolitical factors that influence or mitigate provider burnout, iii) longitudinal changes in burnout in response to extrinsic (i.e. monetary or training) and intrinsic motivational factors, iv) association between burnout, depression, lack of motivation, and other psychological stressors, and v) interventions likely to reduce the burden of burnout. These studies can guide health and policy makers on strategies to improve provider efficiency, productivity, quality, and possibly retention in the workforce.

## Conclusions

The delivery of high quality care in low and middle-income countries requires a workforce that is competent, effective, and motivated. Our results show that provider burnout is prevalent across different cadres of providers in various countries with different health care systems. As we move beyond the Health Workforce Decade (2006–2015) ^61^, towards achieving universal health coverage and the Sustainable Development Goals, populations and countries will require a robust primary health care system to deliver efficient care. Furthermore, the Global Health Workforce Alliance, which was passed at World Health Assembly 2016, specifically highlights a vision in which: “all people everywhere will have access to a skilled, motivated and supported health worker, within a robust health system” ^62^. However, projections show that the global health workforce shortage is only expected to increase over the coming years. In this context, our results suggesting high rates of provider burnout in a number of low and middle income countries underscore the urgent need for health and policy makers to characterize specific risk factors and develop evidence-based interventions to reduce provider burnout, slow down the ongoing attrition of providers from the global workforce, and ensure all patients everywhere receive quality care from motivated and hopeful frontline providers.

## Supplementary material

**Supplementary Materials S1 and S2:** Search terms used in electronic database search.

**Supplementary File 2:** PRISMA checklist.

**Supplementary File 3:** PRISMA flowchart, showing the number of records identified, included and excluded.
