# Supplementary material for: Prevalence and factors associated with burnout among frontline primary health care providers in low- and middle-income countries: A systematic review
Source: Gates Open Res. 2018 Jun 11;2:4. Originally published 2018 Jan 18. [Version 3] doi: 10.12688/gatesopenres.12779.3 (PMC6030396; doi:10.12688/gatesopenres.12779.3)
Supplement: Supplementary file 1 [file gatesopenres-2-13905-s0000.tgz › 5abb2b0a-3fda-4cd6-9ada-f07a5f0ee18b.pdf]

Supplementary Material

S1: Search terms used in electronic database search

EMBASE

('burnout'/de OR 'motivation'/de OR 'achievement'/de OR 'goal attainment'/de OR 'job performance'/exp OR 'depersonalization'/de OR 'job stress'/de OR burnout:ab,ti OR 'burn out':ab,ti OR depersonaliz*:ab,ti OR depersonalis*:ab,ti OR 'personal achievement':ab,ti OR 'emotional exhaustion':ab,ti OR motivat*:ab,ti OR 'occupational stress':ab,ti OR 'job stress':ab,ti OR 'job strain':ab,ti OR 'job related stress':ab,ti OR 'work stress':ab,ti OR 'work related stress':ab,ti)

AND

('health personnel attitude'/exp OR 'hospital personnel'/exp OR 'health care personnel'/exp OR 'social worker'/exp OR 'home' NEXT/2 aid*)

AND

('developing country'/exp OR ('less developed' OR 'low income' OR 'middle income' OR 'resource poor' OR 'resource constrained') NEXT/1 (country OR countries OR region OR regions OR settings OR area OR areas) OR 'American Samoa'/exp OR 'Africa'/exp OR 'African Caribbean'/exp OR 'Borneo'/exp OR 'Brunei Darussalam'/exp OR 'Cambodia'/exp OR 'Caribbean Islands'/exp OR 'China'/exp OR 'Chinese'/exp OR 'Cyprus'/exp OR 'Eastern Europe'/exp OR 'Federated States of Micronesia'/exp OR 'Fiji'/exp OR 'Hong Kong'/exp OR 'Indian'/exp OR 'Indonesia'/exp OR 'Iran'/exp OR 'Iraq'/exp OR 'Jordan'/exp OR 'Kazakhstan'/exp OR 'Kiribati'/exp OR 'Korea'/exp OR 'Kyrgyzstan'/exp OR 'Laos'/exp OR 'Latvia'/exp OR 'Lebanon'/exp OR 'Lithuania'/exp OR 'Macao'/exp OR 'Malaysia'/exp OR 'Maldives'/exp OR 'Marshall Islands'/exp OR 'Mexico'/exp OR 'Mongolia'/exp OR 'Myanmar'/exp OR 'Northern Mariana Islands'/exp OR 'Oman'/exp OR 'Palau'/exp OR 'Palestine'/exp OR 'Papua New Guinea'/exp OR 'Philippines'/exp OR 'Samoa'/exp OR 'Sao Tome and Principe'/exp OR 'Solomon Islands'/exp OR 'Somali (people)'/exp OR 'South and Central America'/exp OR 'South Asia'/exp OR 'Syrian Arab Republic'/exp OR 'Taiwan'/exp OR 'Tajikistan'/exp OR 'Thailand'/exp OR 'Timor-Leste'/exp OR 'Tonga'/exp OR 'Turkey (republic)'/exp OR 'Turkmenistan'/exp OR 'United Arab Emirates'/exp OR 'Uzbekistan'/exp OR 'Viet Nam'/exp OR 'Yemen'/exp) AND [english]/lim AND [embase]/lim

CAB Abstracts

"burnout" OR "burn out" OR (("occupational" OR "job" OR "work") NEAR/2 ("stress" OR "strain")) OR depersonaliz* OR depersonalis* OR "personal achievement" OR "emotional exhaustion" OR motivat*

AND

nurse* OR "midwives" OR physician* OR "family doctor*" OR "general practitioner*" OR "social worker*" OR "health worker*" OR "home health aid*" OR "home care aid*" OR "physical therapist*" OR "health personnel" OR "health provider*" OR "healthcare provider*" OR "care provider*" OR "medical residen*" OR "house staff" OR "fieldworker*" OR "field worker*"

AND

"developing countr*" OR "under developed countr*" OR (("less developed" OR "low income" OR "middle income" OR "resource poor" OR "resource constrained") NEAR/0 (countr* OR region* OR setting* OR area*)) OR Africa* OR Argentin* OR Brazil* OR China OR Chinese OR Chile* OR "Costa Rica*" OR Ghan* OR India* OR Mexic* OR Paragua* OR Russia* OR Rwanda* OR Venezuela*

MEDLINE

("Burnout, Professional"[Mesh] OR "Motivation"[Mesh:NoExp] OR "Achievement"[Mesh] OR burnout[tw] OR burn out[tw] OR depersonaliz*[tw] OR depersonalis*[tw] OR personal achievement[tw] OR emotional exhaustion[tw] OR motivat*[tiab] OR motivate*[ot] OR occupational stress[tw] OR job stress[tw] OR job strain[tw] OR job related stress[tw] OR work stress[tw] OR work related stress[tw])

AND

("Attitude of Health Personnel"[Mesh:NoExp] OR "Health Personnel"[Mesh] OR "Internship and Residency"[Mesh:NoExp] OR medical residen*[tw] OR interns[tw] OR house staff OR nurses[tw] OR nurse[tw] OR nurse's[tw] OR nursing staff[tw] OR nursing assistants[tw] OR midwives[tw] OR physicians[tw] OR physician[tw] OR physician's[tw] OR family doctor*[tw] OR general practitioner*[tw] OR social worker*[tw] OR community health worker*[tw] OR home health aid*[tw] OR home care aid*[tw] OR physical therapist*[tw] OR health personnel[tw] OR health provider*[tw] OR care provider*[tw] OR healthcare provider*[tw] OR fieldworker*[tw] OR field worker*[tw] OR health personnel[tw])

AND

("Internationality"[Mesh:NoExp] OR "Developing Countries"[Mesh] OR (developing countr*[tw] OR under developed countr*[tw] OR (less developed[tw] OR low income[tw] OR middle income[tw] OR resource poor[tw] OR resource constrained[tw]) AND (country[tw] OR countries[tw] OR region[tw] OR regions[tw] OR settings[tw] OR area[tw] OR areas[tw])) OR "Africa South of the Sahara"[Mesh] OR "Central America"[Mesh] OR "South America"[Mesh] OR "Latin America"[Mesh] OR "Caribbean Region"[Mesh] OR "Mexico"[Mesh] OR "Asia"[Mesh:NoExp] OR "Asia, Central"[Mesh] OR "Asia, Northern"[Mesh] OR "Asia, Southeastern"[Mesh] OR "Asia, Western"[Mesh] OR Afghanistan [tiab] OR Afghan [tiab] OR Albania* [tiab] OR Algeria* [tiab] OR American Samoa* [tiab] OR Angola* [tiab] OR Argentina [tiab] OR Argentinian [tiab] OR Armenia* [tiab] OR Azerbaijan* [tiab] OR Bangladesh* [tiab] OR Barbados [tiab] OR Barbadian [tiab] OR Belarus [tiab] OR Belorussian [tiab] OR Beliz* [tiab] OR Benin* [tiab] OR Bhutan* [tiab] OR Bolivia* [tiab] OR Bosnia [tiab] OR Bosnian* [tiab] OR Herzegovin* [tiab] OR Botswan* [tiab] OR Brazil [tiab] OR Brazilian [tiab] OR Bulgaria* [tiab] OR Burkina Faso [tiab] OR Burkinabe [tiab] OR Burmese [tiab] OR Burund* [tiab] OR Cambodia* [tiab] OR Cameroon* [tiab] OR Cape Verde [tiab] OR Cape Verdean [tiab] OR Central African Republic [tiab] OR Chad [tiab] OR Chadian [tiab] OR Chile [tiab] OR Chilean [tiab] OR China [tiab] OR Chinese [tiab] OR Colombia [tiab] OR Colombian [tiab] OR Comoros [tiab] OR Comorian [tiab] OR Congo [tiab] OR Congolese [tiab] OR Costa Rica [tiab] OR Costa Rican [tiab] OR Côte d'Ivoire [tiab] OR Ivory Coast [tiab] OR Ivorian [tiab] OR Croatia* [tiab] OR Cuba [tiab] OR Cuban [tiab] OR Czech [tiab] OR Djibouti* [tiab] OR Dominica [tiab] OR Dominican [tiab] OR Ecuador* [tiab] OR Egypt [tiab] OR Egyptian [tiab] OR El Salvador [tiab] OR Salvadorian [tiab] OR Guinea [tiab] OR Guinean [tiab] OR Eritrea* [tiab] OR Estonia* [tiab] OR Ethiopia* [tiab] OR Fiji* [tiab] OR Gabon* [tiab] OR Gambia* [tiab] OR Gaza [tiab] OR Georgia [tiab] OR Georgian [tiab] OR Ghana [tiab] OR Ghanaian [tiab] OR Grenad* [tiab] OR Guatemala* [tiab] OR Guinea [tiab] OR Guinean [tiab] OR Guyan* [tiab] OR Haiti* [tiab] OR Hondura* [tiab] OR Hong Kong [tiab] OR Hungar* [tiab] OR India [tiab] OR Indian [tiab] OR Indonesia* [tiab] OR Iran [tiab] OR Iranain [tiab] OR Iraq* [tiab] OR Jamaica* [tiab] OR Jordan [tiab] OR Jordanian [tiab] OR Kazakh* [tiab] OR

Kenya [tiab] OR Kenyan [tiab] OR Kiribati [tiab] OR Korea* [tiab] OR Kyrgyz Republic [tiab] OR Kyrgyzstan [tiab] OR Laos [tiab] OR Laotian [tiab] OR Latvia* [tiab] OR Lebanon [tiab] OR Lebanese [tiab] OR Lesotho [tiab] OR Liberia* [tiab] OR Libya* [tiab] OR Lithuania* [tiab] OR Macedonia* [tiab] OR Madagasca* [tiab] OR Malawi* [tiab] OR Malaysia* [tiab] OR Maldives [tiab] OR Maldivian [tiab] OR Mali [tiab] OR Malian [tiab] OR Marshall Islands [tiab] OR Mauritania* [tiab] OR Mauritius [tiab] OR Mauritian [tiab] OR Mayotte [tiab] OR Mexico [tiab] OR Mexican [tiab] OR Micronesia* [tiab] OR Moldov* [tiab] OR Mongolia* [tiab] OR Morocc* [tiab] OR Mozambique [tiab] OR Mozambican [tiab] OR Myanmar [tiab] OR Namibia* [tiab] OR Nepal* [tiab] OR Nicaragua* [tiab] OR Niger [tiab] OR Nigeria* [tiab] OR Northern Mariana Islands [tiab] OR Oman* [tiab] OR Pakistan* [tiab] OR Palau* [tiab] OR Panama* [tiab] OR Papua New Guinea [tiab] OR Paraguay* [tiab] OR Peru* [tiab] OR Philippine* [tiab] OR Poland [tiab] OR Polish [tiab] OR Romania* [tiab] OR Russia [tiab] OR Russian [tiab] OR Rwanda* [tiab] OR Samoa* [tiab] OR Sao Tome [tiab] OR Sao Tomean [tiab] OR Senegal* [tiab] OR Serbia [tiab] OR Serbia* [tiab] OR Montenegr* [tiab] OR Seychell* [tiab] OR Sierra Leone [tiab] OR Sierra Leonian [tiab] OR Slovak Republic [tiab] OR Slovakian [tiab] OR Solomon Islands [tiab] OR Somali* [tiab] OR South Africa [tiab] OR South African [tiab] OR Sri Lanka [tiab] OR Sri Lankan [tiab] OR Saint Kitts [tiab] OR Saint Lucia [tiab] OR Saint Vincent [tiab] OR Sudan* [tiab] OR Suriname* [tiab] OR Swaziland Or Swazi [tiab] OR Syria [tiab] OR Syrian [tiab] OR Tajikistan [tiab] OR Tajik [tiab] OR Tanzania* [tiab] OR Thailand [tiab] OR Thai [tiab] OR Timor-Leste [tiab] OR Togo* [tiab] OR Tonga* [tiab] OR Trinidad and Tobago [tiab] OR Trinidadian [tiab] OR Tobagonian [tiab] OR Tunisia* [tiab] OR Turk* [tiab] OR Turkmenistan [tiab] OR Uganda* [tiab] OR Ukrain* [tiab] OR Uruguay* [tiab] OR Uzbekistan [tiab] OR Uzbek [tiab] OR Vanuat* [tiab] OR Venezuela* [tiab] OR Vietnam* [tiab] OR West Bank [tiab] OR Yemen* [tiab] OR Zambia* [tiab] OR Zimbabwe*) AND English[lang] NOT ("Animals"[Mesh] NOT "Humans"[Mesh]) NOT (Comment[ptyp] OR Editorial[ptyp] OR Letter[ptyp] OR Case Reports[ptyp] OR News[ptyp])

S2: Results of the second electronic database search

The electronic database search was repeated on January 23, 2016, and 770 articles were identified. Of these 9 articles met final inclusion and exclusion criteria, and 761 were excluded for the following reasons: high income countries (13); not focused on burnout (669); not based on health care providers (7); focused on trainees (8); case reports/reviews/editorials/abstracts/qualitative studies (6); inpatient/non-specific setting (54); not in English (3); and not available online (1).
