# Supplementary figures and images for: Prevalence and factors associated with burnout among frontline primary health care providers in low- and middle-income countries: A systematic review
Source: Gates Open Res. 2018 Jun 11;2:4. Originally published 2018 Jan 18. [Version 3] doi: 10.12688/gatesopenres.12779.3 (PMC6030396; doi:10.12688/gatesopenres.12779.3)

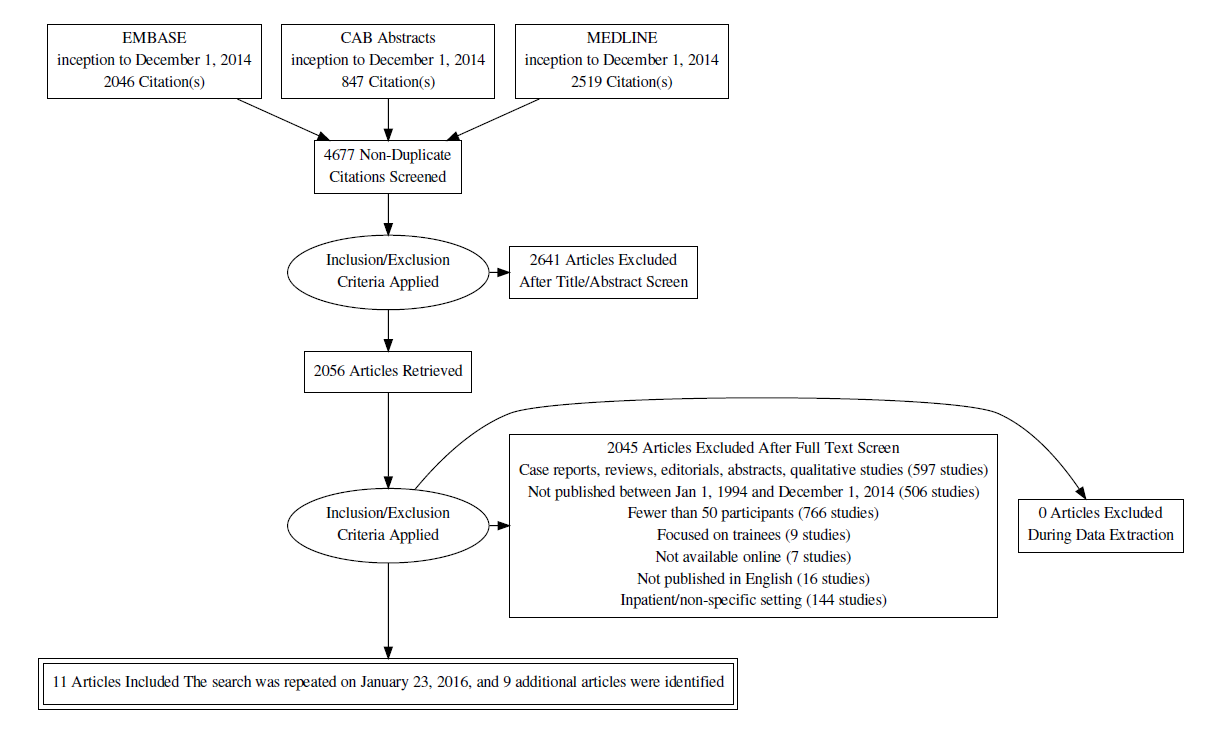

Supplement: Supplementary file 3 [file gatesopenres-2-13905-s0002.tgz › 0dd5f396-6399-4650-b05d-f4bb68774752.png]
