# Supplementary material for: Prevalence and factors associated with burnout among frontline primary health care providers in low- and middle-income countries: A systematic review
Source: Gates Open Res. 2018 Jun 11;2:4. Originally published 2018 Jan 18. [Version 3] doi: 10.12688/gatesopenres.12779.3 (PMC6030396; doi:10.12688/gatesopenres.12779.3)
Supplement: Peer review history

# Peer review: Referee response for version 3

**DOI:** 10.21956/gatesopenres.13905.r26512

**Published:** 2018-06-14

**Authors:** Charlotte Hanlon, Medhin Selamu

**Affiliations:** 1 Institute of Psychology, Psychiatry and Neuroscience, King's College London, London, UK; 2 Addis Ababa University, Addis Ababa, Ethiopia

The authors have now accommodated our comments adequately.

 In the first paragraph of the discussion, we recommend that the authors are less definitive when comparing the level of burnout in LMICs to HICs, given that the meaning of cut-offs for non-validated measures is not known.

We have read this submission. We believe that we have an appropriate level of expertise to confirm that it is of an acceptable scientific standard.

# Peer review: Referee response for version 3

**DOI:** 10.21956/gatesopenres.13905.r26513

**Published:** 2018-06-13

**Authors:** Eliudi Eliakimu

**Affiliations:** 1 Health Quality Assurance Division, Ministry of Health, Community Development, Gender, Elderly and Children, Dar es Salaam, Tanzania

I have no further comments to make on the revised version. ^1^

I have read this submission. I believe that I have an appropriate level of expertise to confirm that it is of an acceptable scientific standard.

## References

[1] DuganiSAfariHHirschhornLRatcliffeHVeillardJMartinGLagomarsinoGBasuLBittonA: Prevalence and factors associated with burnout among frontline primary health care providers in low- and middle-income countries: A systematic review. *Gates Open Research*.2018;2: 10.12688/gatesopenres.12779.3 10.12688/gatesopenres.12779.3 29984356PMC6030396

# Peer review: Referee response for version 2

**DOI:** 10.21956/gatesopenres.13890.r26414

**Published:** 2018-05-11

**Authors:** Charlotte Hanlon, Medhin Selamu

**Affiliations:** 1 Institute of Psychology, Psychiatry and Neuroscience, King's College London, London, UK; 2 Addis Ababa University, Addis Ababa, Ethiopia

**Original review major comments, point 1: **PRISMA flow chart: requires revision 

**Comment 1**:

 The numbers are inconsistent between the abstract and the PRISMA flow chart.

 The reason for excluding studies (and the numbers in each category have not been given.

**Original review major comments, point 2: **There is almost no consideration of the quality of the studies included in the review.  

**Comment 2**:

 The authors have not adequately addressed this comment. The limitation of researchers applying non-validated cut-offs across diverse cultures and settings is not considered at all, even though this threatens the validity of the findings (and is the major problem with the research to date).

 The authors have not conducted any standardised evaluation of the quality of the studies. It if was not done then it needs to be acknowledged as a limitation of this review.

**Original review major comments, point 3: **The authors excluded studies that only focused on healthcare provider mental health without considering burnout, but one criticism of the burnout concept is that it may represent undetected depression (there is a high level of overlap with depression symptoms). There is, therefore, a need for studies that measure both burnout and depression in order to understand the extent to which these constructs are separate. The lack of studies to do this is worth mentioning. 

**Comment 3**: This has not been commented upon in the article.

**Original review major comments, point 6: **Given that all studies were cross-sectional, please speak about ‘associated factors’ rather than risk factors (as we do not know the temporal relationship). There is some inconsistency in the terminology used.

**Comment 4: **This has been addressed adequately.

**Original review major comments, point 8: **The range of health worker cadres included are very diverse in their training level, their role in primary health care, their level of control over their job as well as community’s attitude and respect towards them. Aggregating these heterogeneous groups need to be done with caution. Please can you give more emphasis to the differences among these types of primary healthcare worker. This is also relevant to the discussion, where you talk about modifiable risk factors, but the cadre of health worker to which this pertains is not clear.

**Comment 5**: This has been addressed to some extent.  

**Original review minor comments, point 10: **Please align the conclusion in the abstract and the main paper.  

**Comment 6**: This has been addressed adequately.

**Original review minor comments, point 8: **Please could you make the aim of the review more focused, e.g. to describe the prevalence of, and factors associated with, outpatient provider burnout in LMICs. Then you can comment that you hope that this will then “help managers and policymakers develop and implement effective interventions to prevent burnout and improve work productivity, efficiency, quality and retention.”, but this latter goal seems to be beyond the scope of the review.

**Comment 7**:  this has been addressed adequately.

We have read this submission. We believe that we have an appropriate level of expertise to confirm that it is of an acceptable scientific standard, however we have significant reservations, as outlined above.

## References

[1] DuganiSAfariHHirschhornLRatcliffeHVeillardJMartinGLagomarsinoGBasuLBittonA: Prevalence and factors associated with burnout among frontline primary health care providers in low- and middle-income countries: A systematic review. *Gates Open Research*.2018;2: 10.12688/gatesopenres.12779.2 10.12688/gatesopenres.12779.2 29984356PMC6030396

# Response

**Published:** 2018-06-04

**Authors:** Hannah Ratcliffe

We are grateful for the thoughtful feedback. We have addressed your specific points and have revised the manuscript accordingly. Additional details are provided comment-by-comment, below.

   

***Comment 1:*** *The numbers are inconsistent between the abstract and the PRISMA flow chart. The reason for excluding studies (and the numbers in each category have not been given.*

 The literature search was initially done on December 1, 2014. At that time, 5412 unique articles were identified. The search was repeated on January 23, 2016, and 770 articles were identified. This gives a total of 6,182 articles, which is included in the abstract. In the results section, we have modified the sentence as follows: “…we identified 770 articles (total of 6,182 articles when combined with search on December 1, 2014)”

 In many published articles, reasons for excluding articles are listed in the secondary screening stage and not necessarily at the primary screening stage. Accordingly, we have provided reasons (and number of articles in each case) for excluding articles.   

***Comment 2:*** *The authors have not adequately addressed this comment. The limitation of researchers applying non-validated cut-offs across diverse cultures and settings is not considered at all, even though this threatens the validity of the findings (and is the major problem with the research to date). The authors have not conducted any standardised evaluation of the quality of the studies. It if was not done then it needs to be acknowledged as a limitation of this review.*

 The ‘Discussion’ section has been revised to reflect our evaluation of the quality of the studies.

***Original review major comments, point 3:*** *The authors excluded studies that only focused on healthcare provider mental health without considering burnout, but one criticism of the burnout concept is that it may represent undetected depression (there is a high level of overlap with depression symptoms). There is, therefore, a need for studies that measure both burnout and depression in order to understand the extent to which these constructs are separate. The lack of studies to do this is worth mentioning.*

***Comment 3:*** *This has not been commented upon in the article.*

 We have modified the Discussion section with the following sentence “Further, provider depression may be a cause or consequence of provider burnout, and studies will be required to explore if they are separate or linked constructs”

***Original review major comments, point 8:*** *The range of health worker cadres included are very diverse in their training level, their role in primary health care, their level of control over their job as well as community’s attitude and respect towards them. Aggregating these heterogeneous groups need to be done with caution. Please can you give more emphasis to the differences among these types of primary healthcare worker. This is also relevant to the discussion, where you talk about modifiable risk factors, but the cadre of health worker to which this pertains is not clear.*

***Comment 5:*** *This has been addressed to some extent.*

 We have modified the Discussion section with the following sentence “The roles and responsibilities of different cadres of health workers depends on the study, and therefore should be interpreted carefully”.

# Peer review: Referee response for version 2

**DOI:** 10.21956/gatesopenres.13890.r26415

**Published:** 2018-05-08

**Authors:** Eliudi Eliakimu

**Affiliations:** 1 Health Quality Assurance Division, Ministry of Health, Community Development, Gender, Elderly and Children, Dar es Salaam, Tanzania

The authors have done good job in addressing all the comments raised in my previous report. ^1^ Only a minor error has remained in page 4 of 23 in which they have not changed the study area for reference number 50 (in the version 2) ^2^ to be Zambia instead of Zimbabwe as shown in the quote below:

 " ***Non-MBI measures of provider burnout***

*Four studies used non-MBI tools to measure burnout among providers. A study based in Serbia used a self-assessment test (15 questions assessed on a Freudenberg scale) and reported that 44.4% of community pharmacists had high levels of burnout ^51^. A study based in Zimbabwe used a single question to assess burnout and reported that 27.8% of HIV healthcare providers had burnout that was severe or not improving with time ^50^. In Uganda*,............"

 Apart from the minor error above, I do not have further comments.

I have read this submission. I believe that I have an appropriate level of expertise to confirm that it is of an acceptable scientific standard.

## References

[1] EliakimuE.: Referee Report For: Prevalence and factors associated with burnout among frontline primary health care providers in low- and middle-income countries: A systematic review [version 2; referees: 1 approved, 1 approved with reservations]. *Gates Open Res*.2018;2(4) : 10.21956/gatesopenres.13839.r26231 10.21956/gatesopenres.13839.r26231 29984356PMC6030396

[2] DuganiSAfariHHirschhornLR et al: Prevalence and factors associated with burnout among frontline primary health care providers in low- and middle-income countries: A systematic review [version 2; referees: 1 approved, 1 approved with reservations]. *Gates Open Research*.2018;2(4) : 10.12688/gatesopenres.12779.2 10.12688/gatesopenres.12779.2 29984356PMC6030396

# Response

**Published:** 2018-06-04

**Authors:** Hannah Ratcliffe

Thank you for catching this error--we have updated the revised manuscript accordingly.

# Peer review: Referee response for version 1

**DOI:** 10.21956/gatesopenres.13839.r26227

**Published:** 2018-02-21

**Authors:** Charlotte Hanlon, Medhin Selamu

**Affiliations:** 1 Institute of Psychology, Psychiatry and Neuroscience, King's College London, London, UK; 2 Addis Ababa University, Addis Ababa, Ethiopia

This review is timely in view of the increasing focus on the wellbeing of health workers worldwide and the implications for health systems. We have some suggestions to improve the quality of the paper.

**Major comments**

1. Please include the PRISMA flow chart of studies in the main paper (also, the quality of chart is not good in the supplementary file and it is difficult to read). There is inconsistency between the text in the main paper describing inclusion/exclusion criteria and the flow chart e.g. qualitative studies, the time period of consideration.
2. There is almost no consideration of the quality of the studies included within the review. A key limitation of the research in the area of burnout has been the use of non-validated questionnaires and uncritical application of cut-offs from Western settings to define burnout. At present this review propagates this problem. We appreciate that the authors can only present the data presented in the original papers, but there needs to be critical reflection on the limitation of using cut-offs from non-validated measures. Other aspects of research quality are also crucial. How was social desirability minimised? (to what extent did studies ensure anonymity of responses to allow primary care workers to honestly disclose their feelings?). How representative were the samples? (did they only include health workers who were at work and selectively under-sample health workers who were absent/off sick?). Please expand your discussion of these important methodological issues and the implications for future studies.
3. The authors excluded studies that only focused on healthcare provider mental health without considering burnout, but one criticism of the burnout concept is that it may represent undetected depression (there is a high level of overlap with depression symptoms). There is, therefore, a need for studies that measure both burnout and depression in order to understand the extent to which these constructs are separate. The lack of studies to do this is worth mentioning.
4. Was the review protocol registered (e.g. in Prospero)? If not, this is an important limitation and needs to be acknowledged.
5. It seems surprising that a meta-analysis was not considered, given the high number of studies that used the same assessment measure for burnout. Given our concerns about the quality of the studies, that is probably a good decision, but it needs better justification within the paper.
6. Given that all studies were cross-sectional, please speak about ‘associated factors’ rather than risk factors (as we do not know the temporal relationship). There is some inconsistency in the terminology used.
7. Only two of the studies appear to have been conducted in low-income countries (the rest are middle-income). This is a relevant discussion point.
8. The range of health worker cadres included are very diverse in their training level, their role in primary health care, their level of control over their job as well as community’s attitude and respect towards them. Aggregating these heterogeneous groups need to be done with caution. Please can you give more emphasis to the differences among these types of primary healthcare worker. This is also relevant to the discussion, where you talk about modifiable risk factors, but the cadre of health worker to which this pertains is not clear.

**Minor comments**

1. In the abstract background (and the introduction), we think that it is also relevant to highlight that staff turnover, quality of care and quality of life are key considerations (not just about efficiency and productivity).
2. In the abstract, it might be helpful to add midwives to the list of providers included.
3. In the last three lines of the abstract results you state that “Higher provider burnout was associated with high job stress, high time pressure and workload, and lack of organizational support.” Please relate this to the specific health worker type where the evidence is present.
4. In the introduction (line 11) it would be helpful if you indicated whether the 50-80% spending on staff remuneration is for low or high income countries.
5. Please add a title for the Methods.
6. Was data extraction done by more than one person independently?
7. At the end of the introduction you have a comment on the paucity of high quality studies on burnout in LMICs, emphasising the cross-sectional nature of studies etc. This is really the finding of your review and doesn’t fit well in the introduction.
8. Please could you make the aim of the review more focused, e.g. to describe the prevalence of, and factors associated with, outpatient provider burnout in LMICs. Then you can comment that you hope that this will then “help managers and policymakers develop and implement effective interventions to prevent burnout and improve work productivity, efficiency, quality and retention.”, but this latter goal seems to be beyond the scope of the review.
9. At the beginning of the results there is some repetition of the cadres of health worker (but inconsistent with one another and different from the tables). Please ensure consistency. Where are the studies including dentists?
10. Please align the conclusion in the abstract and the main paper.

We have read this submission. We believe that we have an appropriate level of expertise to confirm that it is of an acceptable scientific standard, however we have significant reservations, as outlined above.

# Response

**Published:** 2018-04-25

**Authors:** Hannah Ratcliffe

Thank you for your insightful comments and questions. Please see below a detailed explanation of how each of your comments was addressed.

**Major comments**

*1) Please include the PRISMA flow chart of studies in the main paper (also, the quality of chart is not good in the supplementary file and it is difficult to read). There is inconsistency between the text in the main paper describing inclusion/exclusion criteria and the flow chart e.g. qualitative studies, the time period of consideration.* 

Based on the journal’s preferred format, the protocol has been added to the supplementary appendix. The literature search was done twice (December 1, 2014, and January 23, 2016), as explained in the “Data sources and search strategy” section. We have amended the methods section to clarify that qualitative studies were also excluded. We apologize for the inconsistency.

*2)There is almost no consideration of the quality of the studies included within the review. A key limitation of the research in the area of burnout has been the use of non-validated questionnaires and uncritical application of cut-offs from Western settings to define burnout. At present this review propagates this problem. We appreciate that the authors can only present the data presented in the original papers, but there needs to be critical reflection on the limitation of using cut-offs from non-validated measures. Other aspects of research quality are also crucial. How was social desirability minimised? (to what extent did studies ensure anonymity of responses to allow primary care workers to honestly disclose their feelings?). How representative were the samples? (did they only include health workers who were at work and selectively under-sample health workers who were absent/off sick?). Please expand your discussion of these important methodological issues and the implications for future studies.* 

The reviewer raises several excellent points as under-sampling, purposeful sampling, and characteristics of non-responders will dictate the validity of the results. These are important points that should be taken into consideration for future studies. We have added the following to the discussion on Page 14: “Further studies should ensure higher response rates among responders, describe the characteristics of non-responders, and clarify the level of anonymity responders have while participating in studies. Further, as many studies are cross-sectional, it will be challenging to generalize these findings to different contexts and situations.”

*3) The authors excluded studies that only focused on healthcare provider mental health without considering burnout, but one criticism of the burnout concept is that it may represent undetected depression (there is a high level of overlap with depression symptoms). There is, therefore, a need for studies that measure both burnout and depression in order to understand the extent to which these constructs are separate. The lack of studies to do this is worth mentioning.* 

We agree that burnout may represent undetected depression. Further, burnout may be a precursor to depression, result from it, or be worsened by it. Separating burnout, lack of motivation, depression, and other psychologic stressors is important as they may point to different interventional strategies. We agree that further studies should focus on this.

*4) Was the review protocol registered (e.g. in Prospero)? If not, this is an important limitation and needs to be acknowledged.* 

The protocol was not registered, and we have added this on Page 5 under “Data sources and search strategy”

*5) It seems surprising that a meta-analysis was not considered, given the high number of studies that used the same assessment measure for burnout. Given our concerns about the quality of the studies, that is probably a good decision, but it needs better justification within the paper.*

In the discussion section, we address reasons for not considering a meta-analysis. We noted that “only 15 of the 20 included studies used the MBI, and among them, differences in study population and design precluded analyses across different cadres of health providers”.

*6) Given that all studies were cross-sectional, please speak about ‘associated factors’ rather than risk factors (as we do not know the temporal relationship). There is some inconsistency in the terminology used.*

Many studies discuss the risk associated with burnout. We agree that this is association and not causation. In these cross-sectional studies, it is likely that some of the observations result from burnout and may not be a risk associated with developing burnout.

*7) Only two of the studies appear to have been conducted in low-income countries (the rest are middle-income). This is a relevant discussion point.*

We agree and have added the following to the Results section “Two studies were based in low income countries while the rest were based in middle-income countries”.

*8) The range of health worker cadres included are very diverse in their training level, their role in primary health care, their level of control over their job as well as community’s attitude and respect towards them. Aggregating these heterogeneous groups need to be done with caution. Please can you give more emphasis to the differences among these types of primary healthcare worker. This is also relevant to the discussion, where you talk about modifiable risk factors, but the cadre of health worker to which this pertains is not clear.*

We noted a variety of cadres, and included this in the discussion as a factor that precluded doing a meta-analysis. The educational background and qualifications of each cadre vary resulting in different healthcare responsibilities. From the studies, we could not determine these factors.

**Minor comments**

*1)In the abstract background (and the introduction), we think that it is also relevant to highlight that staff turnover, quality of care and quality of life are key considerations (not just about efficiency and productivity).*

We have added the following sentence to the introduction “Further, a positive work environment will reduce workforce turnover, and improve quality of life and care”.

2) *In the abstract, it might be helpful to add midwives to the list of providers included.*

This change has been made

*3) In the last three lines of the abstract results you state that “Higher provider burnout was associated with high job stress, high time pressure and workload, and lack of organizational support.” Please relate this to the specific health worker type where the evidence is present.*We have made the following change “Higher provider burnout (for example, among nurses, pharmacists, and rural health workers) was…”

*4) In the introduction (line 11) it would be helpful if you indicated whether the 50-80% spending on staff remuneration is for low or high income countries.*

We have focused those sentences on retention and quality of life, therefore deleted the section related to salaries.

*5) Please add a title for the Methods.*

This change has been made

*6) Was data extraction done by more than one person independently?*

Yes, as noted in the methods section, all articles were extracted independently by 2 study team members. The method for resolving conflicts is also described.

*7) At the end of the introduction you have a comment on the paucity of high quality studies on burnout in LMICs, emphasising the cross-sectional nature of studies etc. This is really the finding of your review and doesn’t fit well in the introduction.*

We meant that paucity of synthesis of studies on burnout. We have modified this to read “In addition to the paucity of such synthesis, current data…”

*8) Please could you make the aim of the review more focused, e.g. to describe the prevalence of, and factors associated with, outpatient provider burnout in LMICs. Then you can comment that you hope that this will then “help managers and policymakers develop and implement effective interventions to prevent burnout and improve work productivity, efficiency, quality and retention.”, but this latter goal seems to be beyond the scope of the review.*

We agree and have made this change.

*9) At the beginning of the results there is some repetition of the cadres of health worker (but inconsistent with one another and different from the tables). Please ensure consistency. Where are the studies including dentists?*

We have made changes to ensure consistency. We did not include dentists in our study. We strongly think this is a vital cadre and should be the subject of future studies.  

*10) Please align the conclusion in the abstract and the main paper.*

We agree with the reviewer and have made the following changes to the conclusions in the abstract to read “Our comprehensive review of published literature showed that provider burnout is prevalent across various health care providers in LMICs. Further studies are required to better measure the causes and consequences of burnout, and guide the development of effective interventions to reduce or prevent burnout.”

# Peer review: Referee response for version 1

**DOI:** 10.21956/gatesopenres.13839.r26231

**Published:** 2018-02-09

**Authors:** Eliudi Eliakimu

**Affiliations:** 1 Health Quality Assurance Division, Ministry of Health, Community Development, Gender, Elderly and Children, Dar es Salaam, Tanzania

This report provides a review of the paper by Dugani, et al (2018). ^1^ The rationale of the paper by Dugani and colleagues is to fill the knowledge gap regarding the lack of comprehensive analysis of the burden of burnout and contributing factors in low-and middle – income countries (LMICs). The authors have clearly shown their objective for the study – “…..to describe the prevalence of and factors associated with outpatient provider burnout in LMICs, to help managers and policy makers develop and implement effective interventions…..” The authors went on to give clear details of the methodologic approach (search strategy, selection of the studies, data extraction and analysis) and then they presented well their results, discussion and conclusions.

 The purpose of this review report is to vet the paper in terms of whether it is scientifically sound. The report is sequenced into six parts as per the paper layout (introduction, methodology, results, discussion, conclusions, and reference list).

*1. Introduction* – the authors have given clear introduction with description of primary health care system and its key requirement (i.e., availability of skilled and motivated human resources, and they have included projections of human resources shortage); and then they focused the rest of it on burnout. To my expertise, I find the introductory part to be well-done and scientifically sound.

*2. Methodology* – it is well presented with clear details of the search process (sources and strategy) and how the studies were selected by indicating the role played by each of authors. To the best of my knowledge, I approve the methodology used and details provided on the process that they are valid and robust.  

*3. Results* – the results section is well presented and of acceptable standard. However, there are some minor corrections, which the authors need to make on the following:

- On the paragraph under the subheading “Non-MBI measures of provider burnout” and the paragraph under the subheading “variables associated with provider burnout”, the authors have made an error by wrongly referring the study by Kruse, et al (2009), which is reference number 51 in the paper to be from Zimbabwe while it was done in Zambia – Lusaka District. ^2 ^
- In table 1 – Kruse, et al (2009) ^2 ^the same error is seen indicating it was done in Zimbabwe instead of Zambia.
- There is a need for consistency of the names of authors used in the tables 1, 2, and 3 and the way they appear in the reference list. In particular here, I refer to “da Silva (2008)” – in the reference list (number 37) it is cited as “Silva”. In this case, the authors need to find the correct name for the author of reference-37 and use both in the tables and in the reference list in order to enable readers to follow through smoothly.

In terms of how exhaustive the literature search has been, the authors have done a very comprehensive search strategy. However, there could be some literatures that have not been captured. For example, I suggest to the authors to consider inclusion of the paper by Perry, et al (2014) ^3^ on burnout among voluntary medical male circumcision (VMMC) services providers. By nature of the VMMC services, they are usually outpatient in nature.

*4. Discussion *– it is well presented and linked to the results.

*5. Conclusions* – the authors have synthesized well the conclusions and linked it with the results and discussion.

*6. Reference list* – the authors need to ensure correctness of the references both in the text (narrative and in tables) and in the reference list.

I have read this submission. I believe that I have an appropriate level of expertise to confirm that it is of an acceptable scientific standard.

## References

[1] DuganiSAfariHHirschhornL.RRatcliffeHVeillardJMartinGLagomarsinoGBasuLBittonA: Prevalence and factors associated with burnout among frontline primary health care providers in low- and middle-income countries: A systematic review [version 1; referees: awaiting peer review]. *Gates Open Res*.2018;2(4) : 10.12688/gatesopenres.12779.1 10.12688/gatesopenres.12779.1 29984356PMC6030396

[2] KruseGRChapulaBTIkedaSNkhomaMQuiterioNPankratzDMatakaKChiBHBondVReidSE: Burnout and use of HIV services among health care workers in Lusaka District, Zambia: a cross-sectional study. *Hum Resour Health*.2009;7: 10.1186/1478-4491-7-55 55 10.1186/1478-4491-7-55 19594917PMC2714832

[3] PerryLRechDMavhuWFradeSMachakuMDOnyangoMAdudaDSFimboBCherutichPCastorDNjeuhmeliEBertrandJT: Work experience, job-fulfillment and burnout among VMMC providers in Kenya, South Africa, Tanzania and Zimbabwe. *PLoS One*.2014;9(5) : 10.1371/journal.pone.0084215 e84215 10.1371/journal.pone.0084215 24802260PMC4011707

# Response

**Published:** 2018-04-25

**Authors:** Hannah Ratcliffe

Thank you for your insightful comments and questions. Please see below a detailed explanation of how each of your comments was addressed.

*1) On the paragraph under the subheading “Non-MBI measures of provider burnout” and the paragraph under the subheading “variables associated with provider burnout”, the authors have made an error by wrongly referring the study by Kruse, et al (2009), which is reference number 51 in the paper to be from Zimbabwe while it was done in Zambia – Lusaka District. ^2^*

We have corrected this error.

*2) In table 1 – Kruse, et al (2009) ^2 ^the same error is seen indicating it was done in Zimbabwe instead of Zambia.*

We have corrected this error.

*3) There is a need for consistency of the names of authors used in the tables 1, 2, and 3 and the way they appear in the reference list. In particular here, I refer to “da Silva (2008)” – in the reference list (number 37) it is cited as “Silva”. In this case, the authors need to find the correct name for the author of reference-37 and use both in the tables and in the reference list in order to enable readers to follow through smoothly*

We have corrected this error.

*4) In terms of how exhaustive the literature search has been, the authors have done a very comprehensive search strategy. However, there could be some literatures that have not been captured. For example, I suggest to the authors to consider inclusion of the paper by Perry, et al (2014) ^3^ on burnout among voluntary medical male circumcision (VMMC) services providers. By nature of the VMMC services, they are usually outpatient in nature.*We thank the reviewer for suggesting this paper.

We had encountered this paper, but excluded it as the setting (outpatient vs. inpatient) was unclear to us. As a result we will exclude it from our manuscript.

*5) Reference list – the authors need to ensure correctness of the references both in the text (narrative and in tables) and in the reference list.*

We have reviewed the references.
